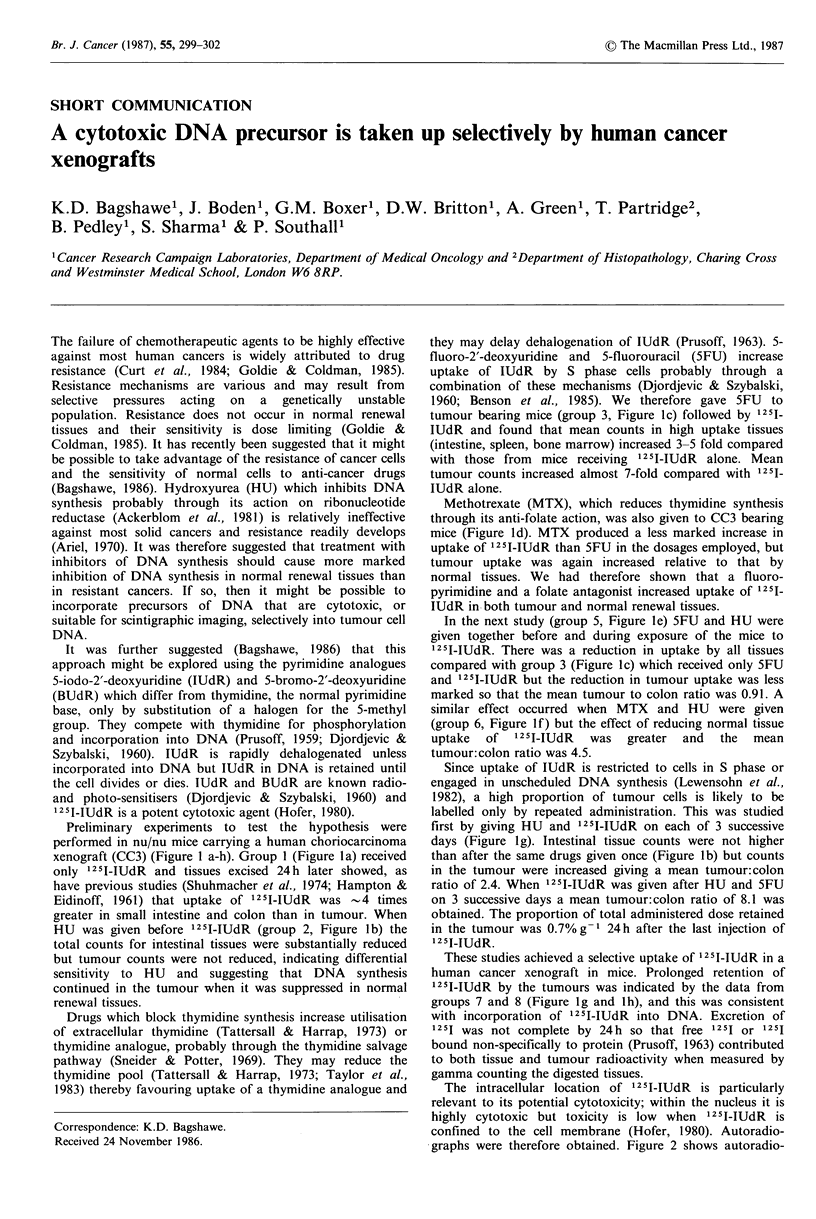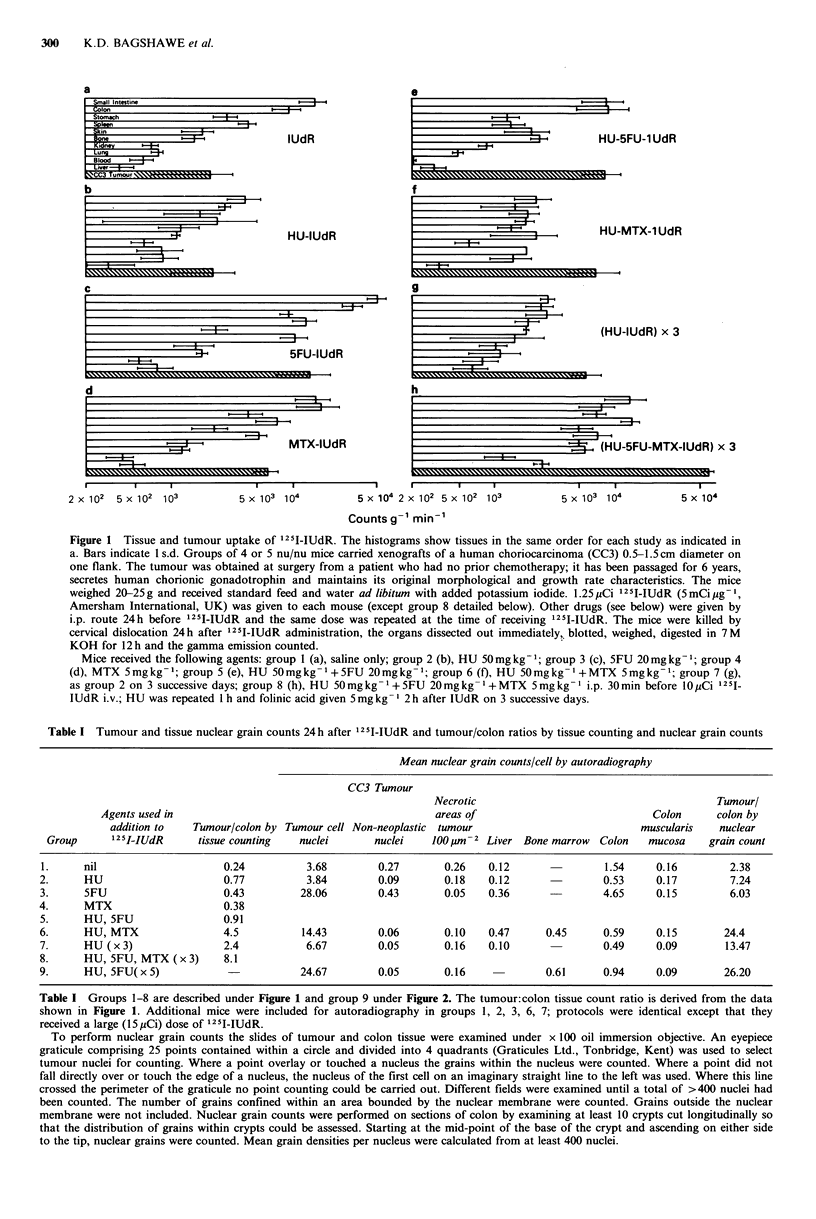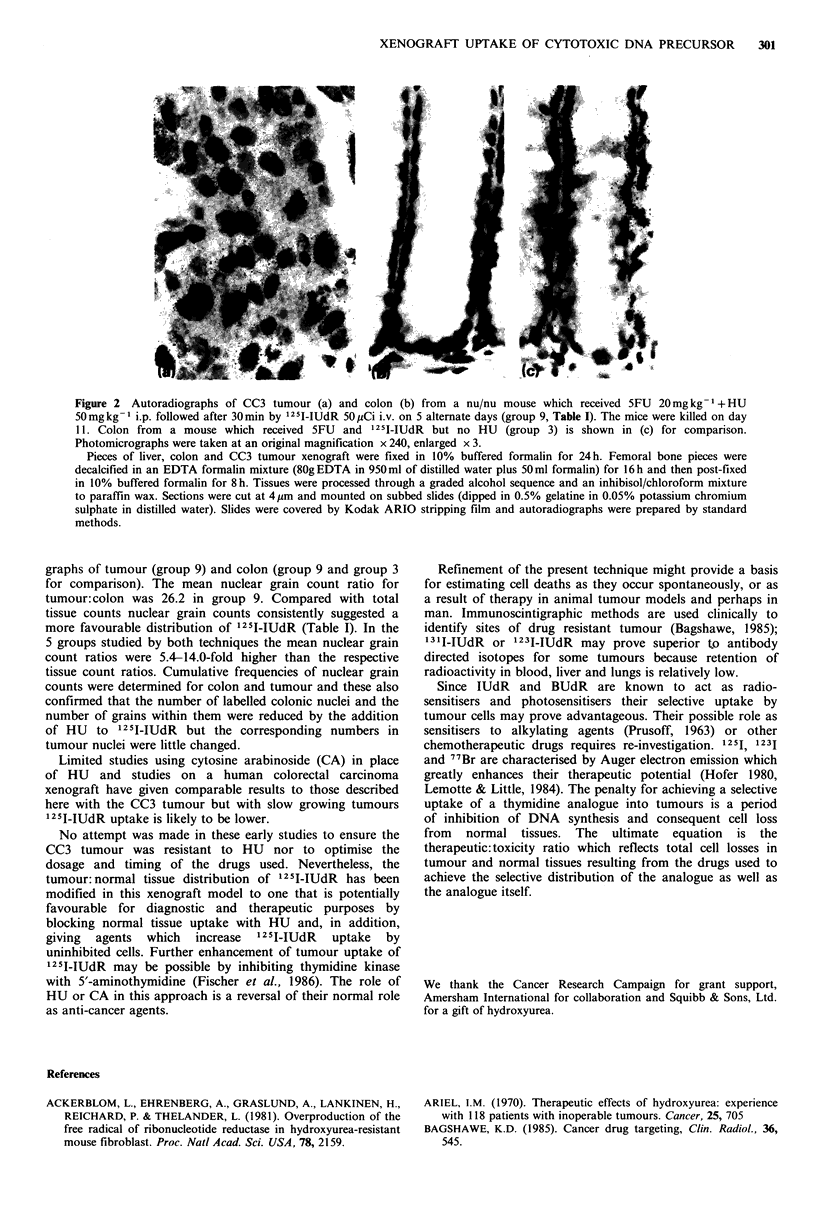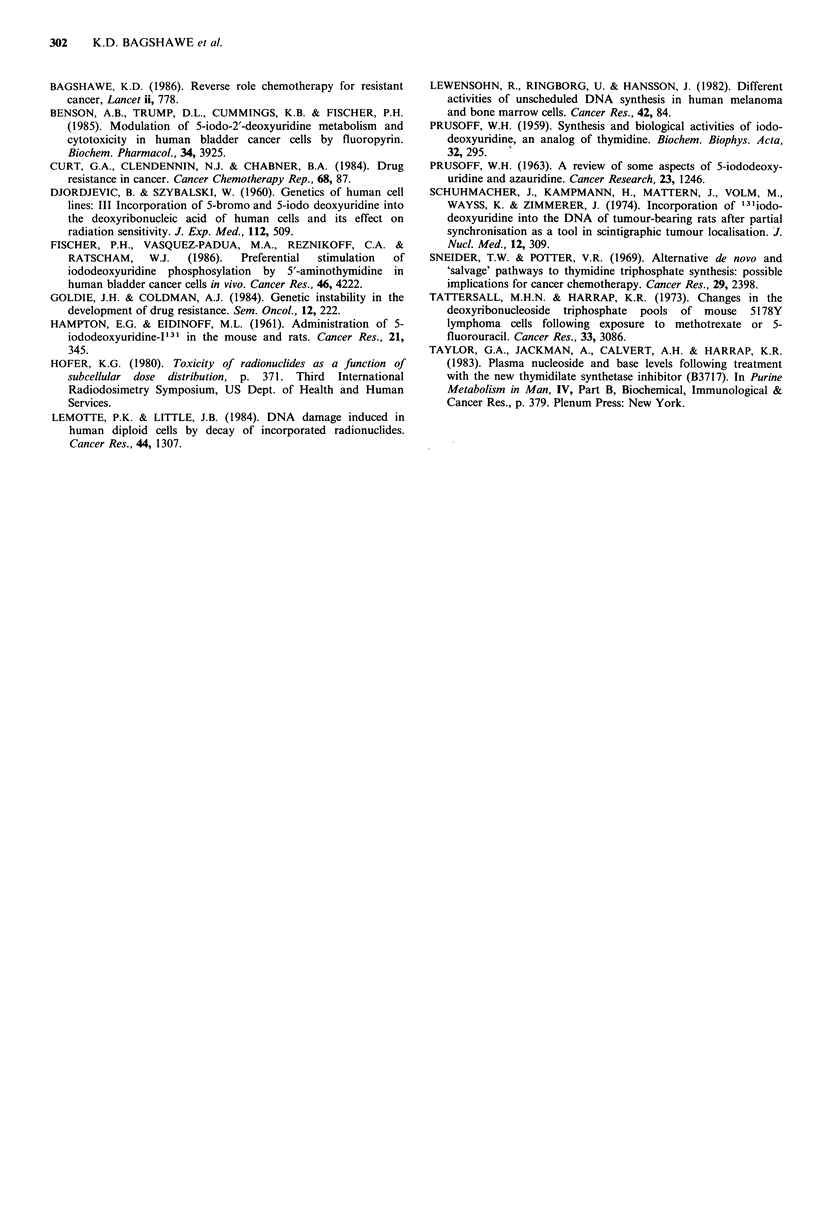# A cytotoxic DNA precursor is taken up selectively by human cancer xenografts.

**DOI:** 10.1038/bjc.1987.58

**Published:** 1987-03

**Authors:** K. D. Bagshawe, J. Boden, G. M. Boxer, D. W. Britton, A. Green, T. Partridge, B. Pedley, S. Sharma, P. Southall

## Abstract

**Images:**


*Br. J. Cancer* (1987), **55**, 299–302 © The Macmillan Press Ltd., 1987

SHORT COMMUNICATION

### A cytotoxic DNA precursor is taken up selectively by human cancer xenografts

K.D. Bagshawe[1], J. Boden[1], G.M. Boxer[1], D.W. Britton[1], A. Green[1], T. Partridge[2], B. Pedley[1], S. Sharma[1] & P. Southall[1]

[1]*Cancer Research Campaign Laboratories, Department of Medical Oncology and* [2]*Department of Histopathology, Charing Cross and Westminster Medical School, London W6 8RP.*

The failure of chemotherapeutic agents to be highly effective against most human cancers is widely attributed to drug resistance (Curt *et al.*, 1984; Goldie & Coldman, 1985). Resistance mechanisms are various and may result from selective pressures acting on a genetically unstable population. Resistance does not occur in normal renewal tissues and their sensitivity is dose limiting (Goldie & Coldman, 1985). It has recently been suggested that it might be possible to take advantage of the resistance of cancer cells and the sensitivity of normal cells to anti-cancer drugs (Bagshawe, 1986). Hydroxyurea (HU) which inhibits DNA synthesis probably through its action on ribonucleotide reductase (Ackerblom *et al.*, 1981) is relatively ineffective against most solid cancers and resistance readily develops (Ariel, 1970). It was therefore suggested that treatment with inhibitors of DNA synthesis should cause more marked inhibition of DNA synthesis in normal renewal tissues than in resistant cancers. If so, then it might be possible to incorporate precursors of DNA that are cytotoxic, or suitable for scintigraphic imaging, selectively into tumour cell DNA.

It was further suggested (Bagshawe, 1986) that this approach might be explored using the pyrimidine analogues 5-iodo-2′-deoxyuridine (IUdR) and 5-bromo-2′-deoxyuridine (BUdR) which differ from thymidine, the normal pyrimidine base, only by substitution of a halogen for the 5-methyl group. They compete with thymidine for phosphorylation and incorporation into DNA (Prusoff, 1959; Djordjevic & Szybalski, 1960). IUdR is rapidly dehalogenated unless incorporated into DNA but IUdR in DNA is retained until the cell divides or dies. IUdR and BUdR are known radio- and photo-sensitisers (Djordjevic & Szybalski, 1960) and $^{125}$I-IUdR is a potent cytotoxic agent (Hofer, 1980).

Preliminary experiments to test the hypothesis were performed in nu/nu mice carrying a human choriocarcinoma xenograft (CC3) (Figure 1 a-h). Group 1 (Figure 1a) received only $^{125}$I-IUdR and tissues excised 24 h later showed, as have previous studies (Shuhmacher *et al.*, 1974; Hampton & Eidinoff, 1961) that uptake of $^{125}$I-IUdR was $\sim$4 times greater in small intestine and colon than in tumour. When HU was given before $^{125}$I-IUdR (group 2, Figure 1b) the total counts for intestinal tissues were substantially reduced but tumour counts were not reduced, indicating differential sensitivity to HU and suggesting that DNA synthesis continued in the tumour when it was suppressed in normal renewal tissues.

Drugs which block thymidine synthesis increase utilisation of extracellular thymidine (Tattersall & Harrap, 1973) or thymidine analogue, probably through the thymidine salvage pathway (Sneider & Potter, 1969). They may reduce the thymidine pool (Tattersall & Harrap, 1973; Taylor *et al.*, 1983) thereby favouring uptake of a thymidine analogue and they may delay dehalogenation of IUdR (Prusoff, 1963). 5-fluoro-2′-deoxyuridine and 5-fluorouracil (5FU) increase uptake of IUdR by S phase cells probably through a combination of these mechanisms (Djordjevic & Szybalski, 1960; Benson *et al.*, 1985). We therefore gave 5FU to tumour bearing mice (group 3, Figure 1c) followed by $^{125}$I-IUdR and found that mean counts in high uptake tissues (intestine, spleen, bone marrow) increased 3–5 fold compared with those from mice receiving $^{125}$I-IUdR alone. Mean tumour counts increased almost 7-fold compared with $^{125}$I-IUdR alone.

Methotrexate (MTX), which reduces thymidine synthesis through its anti-folate action, was also given to CC3 bearing mice (Figure 1d). MTX produced a less marked increase in uptake of $^{125}$I-IUdR than 5FU in the dosages employed, but tumour uptake was again increased relative to that by normal tissues. We had therefore shown that a fluoropyrimidine and a folate antagonist increased uptake of $^{125}$I-IUdR in both tumour and normal renewal tissues.

In the next study (group 5, Figure 1e) 5FU and HU were given together before and during exposure of the mice to $^{125}$I-IUdR. There was a reduction in uptake by all tissues compared with group 3 (Figure 1c) which received only 5FU and $^{125}$I-IUdR but the reduction in tumour uptake was less marked so that the mean tumour to colon ratio was 0.91. A similar effect occurred when MTX and HU were given (group 6, Figure 1f) but the effect of reducing normal tissue uptake of $^{125}$I-IUdR was greater and the mean tumour:colon ratio was 4.5.

Since uptake of IUdR is restricted to cells in S phase or engaged in unscheduled DNA synthesis (Lewensohn *et al.*, 1982), a high proportion of tumour cells is likely to be labelled only by repeated administration. This was studied first by giving HU and $^{125}$I-IUdR on each of 3 successive days (Figure 1g). Intestinal tissue counts were not higher than after the same drugs given once (Figure 1b) but counts in the tumour were increased giving a mean tumour:colon ratio of 2.4. When $^{125}$I-IUdR was given after HU and 5FU on 3 successive days a mean tumour:colon ratio of 8.1 was obtained. The proportion of total administered dose retained in the tumour was 0.7% g$^{-1}$ 24 h after the last injection of $^{125}$I-IUdR.

These studies achieved a selective uptake of $^{125}$I-IUdR in a human cancer xenograft in mice. Prolonged retention of $^{125}$I-IUdR by the tumours was indicated by the data from groups 7 and 8 (Figure 1g and 1h), and this was consistent with incorporation of $^{125}$I-IUdR into DNA. Excretion of $^{125}$I was not complete by 24 h so that free $^{125}$I or $^{125}$I bound non-specifically to protein (Prusoff, 1963) contributed to both tissue and tumour radioactivity when measured by gamma counting the digested tissues.

The intracellular location of $^{125}$I-IUdR is particularly relevant to its potential cytotoxicity; within the nucleus it is highly cytotoxic but toxicity is low when $^{125}$I-IUdR is confined to the cell membrane (Hofer, 1980). Autoradiographs were therefore obtained. Figure 2 shows autoradio-

Correspondence: K.D. Bagshawe.
Received 24 November 1986.

**Figure 1** Tissue and tumour uptake of $^{125}$I-IUdR. The histograms show tissues in the same order for each study as indicated in a. Bars indicate 1 s.d. Groups of 4 or 5 nu/nu mice carried xenografts of a human choriocarcinoma (CC3) 0.5–1.5 cm diameter on one flank. The tumour was obtained at surgery from a patient who had no prior chemotherapy; it has been passaged for 6 years, secretes human chorionic gonadotrophin and maintains its original morphological and growth rate characteristics. The mice weighed 20–25 g and received standard feed and water *ad libitum* with added potassium iodide. 1.25 $\mu$Ci $^{125}$I-IUdR (5 mCi $\mu g^{-1}$, Amersham International, UK) was given to each mouse (except group 8 detailed below). Other drugs (see below) were given by i.p. route 24 h before $^{125}$I-IUdR and the same dose was repeated at the time of receiving $^{125}$I-IUdR. The mice were killed by cervical dislocation 24 h after $^{125}$I-IUdR administration, the organs dissected out immediately, blotted, weighed, digested in 7 M KOH for 12 h and the gamma emission counted.

Mice received the following agents: group 1 (a), saline only; group 2 (b), HU 50 mg kg$^{-1}$; group 3 (c), 5FU 20 mg kg$^{-1}$; group 4 (d), MTX 5 mg kg$^{-1}$; group 5 (e), HU 50 mg kg$^{-1}$ + 5FU 20 mg kg$^{-1}$; group 6 (f), HU 50 mg kg$^{-1}$ + MTX 5 mg kg$^{-1}$; group 7 (g), as group 2 on 3 successive days; group 8 (h), HU 50 mg kg$^{-1}$ + 5FU 20 mg kg$^{-1}$ + MTX 5 mg kg$^{-1}$ i.p. 30 min before 10 $\mu$Ci $^{125}$I-IUdR i.v.; HU was repeated 1 h and folinic acid given 5 mg kg$^{-1}$ 2 h after IUdR on 3 successive days.

**Table I** Tumour and tissue nuclear grain counts 24 h after $^{125}$I-IUdR and tumour/colon ratios by tissue counting and nuclear grain counts

| | | | *Mean nuclear grain counts/cell by autoradiography* | | | | | | | |
|---|---|---|---|---|---|---|---|---|---|---|
| | | | *CC3 Tumour* | | | | | | | |
| *Group* | *Agents used in addition to $^{125}$I-IUdR* | *Tumour/colon by tissue counting* | *Tumour cell nuclei* | *Non-neoplastic nuclei* | *Necrotic areas of tumour 100 $\mu m^{-2}$* | *Liver* | *Bone marrow* | *Colon* | *Colon muscularis mucosa* | *Tumour/colon by nuclear grain count* |
| 1. | nil | 0.24 | 3.68 | 0.27 | 0.26 | 0.12 | — | 1.54 | 0.16 | 2.38 |
| 2. | HU | 0.77 | 3.84 | 0.09 | 0.18 | 0.12 | — | 0.53 | 0.17 | 7.24 |
| 3. | 5FU | 0.43 | 28.06 | 0.43 | 0.05 | 0.36 | — | 4.65 | 0.15 | 6.03 |
| 4. | MTX | 0.38 | | | | | | | | |
| 5. | HU, 5FU | 0.91 | | | | | | | | |
| 6. | HU, MTX | 4.5 | 14.43 | 0.06 | 0.10 | 0.47 | 0.45 | 0.59 | 0.15 | 24.4 |
| 7. | HU (×3) | 2.4 | 6.67 | 0.05 | 0.16 | 0.10 | — | 0.49 | 0.09 | 13.47 |
| 8. | HU, 5FU, MTX (×3) | 8.1 | | | | | | | | |
| 9. | HU, 5FU(×5) | — | 24.67 | 0.05 | 0.16 | — | 0.61 | 0.94 | 0.09 | 26.20 |

**Table I** Groups 1–8 are described under **Figure 1** and group 9 under **Figure 2.** The tumour:colon tissue count ratio is derived from the data shown in **Figure 1.** Additional mice were included for autoradiography in groups 1, 2, 3, 6, 7; protocols were identical except that they received a large (15 $\mu$Ci) dose of $^{125}$I-IUdR.

To perform nuclear grain counts the slides of tumour and colon tissue were examined under ×100 oil immersion objective. An eyepiece graticule comprising 25 points contained within a circle and divided into 4 quadrants (Graticules Ltd., Tonbridge, Kent) was used to select tumour nuclei for counting. Where a point overlay or touched a nucleus the grains within the nucleus were counted. Where a point did not fall directly over or touch the edge of a nucleus, the nucleus of the first cell on an imaginary straight line to the left was used. Where this line crossed the perimeter of the graticule no point counting could be carried out. Different fields were examined until a total of >400 nuclei had been counted. The number of grains confined within an area bounded by the nuclear membrane were counted. Grains outside the nuclear membrane were not included. Nuclear grain counts were performed on sections of colon by examining at least 10 crypts cut longitudinally so that the distribution of grains within crypts could be assessed. Starting at the mid-point of the base of the crypt and ascending on either side to the tip, nuclear grains were counted. Mean grain densities per nucleus were calculated from at least 400 nuclei.

**Figure 2** Autoradiographs of CC3 tumour (a) and colon (b) from a nu/nu mouse which received 5FU 20 mg kg$^{-1}$ + HU 50 mg kg$^{-1}$ i.p. followed after 30 min by $^{125}$I-IUdR 50 $\mu$Ci i.v. on 5 alternate days (group 9, **Table I**). The mice were killed on day 11. Colon from a mouse which received 5FU and $^{125}$I-IUdR but no HU (group 3) is shown in (c) for comparison. Photomicrographs were taken at an original magnification ×240, enlarged ×3.

Pieces of liver, colon and CC3 tumour xenograft were fixed in 10% buffered formalin for 24 h. Femoral bone pieces were decalcified in an EDTA formalin mixture (80g EDTA in 950 ml of distilled water plus 50 ml formalin) for 16 h and then post-fixed in 10% buffered formalin for 8 h. Tissues were processed through a graded alcohol sequence and an inhibisol/chloroform mixture to paraffin wax. Sections were cut at 4 $\mu$m and mounted on subbed slides (dipped in 0.5% gelatine in 0.05% potassium chromium sulphate in distilled water). Slides were covered by Kodak ARIO stripping film and autoradiographs were prepared by standard methods.

graphs of tumour (group 9) and colon (group 9 and group 3 for comparison). The mean nuclear grain count ratio for tumour:colon was 26.2 in group 9. Compared with total tissue counts nuclear grain counts consistently suggested a more favourable distribution of $^{125}$I-IUdR (Table I). In the 5 groups studied by both techniques the mean nuclear grain count ratios were 5.4–14.0-fold higher than the respective tissue count ratios. Cumulative frequencies of nuclear grain counts were determined for colon and tumour and these also confirmed that the number of labelled colonic nuclei and the number of grains within them were reduced by the addition of HU to $^{125}$I-IUdR but the corresponding numbers in tumour nuclei were little changed.

Limited studies using cytosine arabinoside (CA) in place of HU and studies on a human colorectal carcinoma xenograft have given comparable results to those described here with the CC3 tumour but with slow growing tumours $^{125}$I-IUdR uptake is likely to be lower.

No attempt was made in these early studies to ensure the CC3 tumour was resistant to HU nor to optimise the dosage and timing of the drugs used. Nevertheless, the tumour: normal tissue distribution of $^{125}$I-IUdR has been modified in this xenograft model to one that is potentially favourable for diagnostic and therapeutic purposes by blocking normal tissue uptake with HU and, in addition, giving agents which increase $^{125}$I-IUdR uptake by uninhibited cells. Further enhancement of tumour uptake of $^{125}$I-IUdR may be possible by inhibiting thymidine kinase with 5′-aminothymidine (Fischer *et al.*, 1986). The role of HU or CA in this approach is a reversal of their normal role as anti-cancer agents.

Refinement of the present technique might provide a basis for estimating cell deaths as they occur spontaneously, or as a result of therapy in animal tumour models and perhaps in man. Immunoscintigraphic methods are used clinically to identify sites of drug resistant tumour (Bagshawe, 1985); $^{131}$I-IUdR or $^{123}$I-IUdR may prove superior to antibody directed isotopes for some tumours because retention of radioactivity in blood, liver and lungs is relatively low.

Since IUdR and BUdR are known to act as radio-sensitisers and photosensitisers their selective uptake by tumour cells may prove advantageous. Their possible role as sensitisers to alkylating agents (Prusoff, 1963) or other chemotherapeutic drugs requires re-investigation. $^{125}$I, $^{123}$I and $^{77}$Br are characterised by Auger electron emission which greatly enhances their therapeutic potential (Hofer 1980, Lemotte & Little, 1984). The penalty for achieving a selective uptake of a thymidine analogue into tumours is a period of inhibition of DNA synthesis and consequent cell loss from normal tissues. The ultimate equation is the therapeutic:toxicity ratio which reflects total cell losses in tumour and normal tissues resulting from the drugs used to achieve the selective distribution of the analogue as well as the analogue itself.

We thank the Cancer Research Campaign for grant support, Amersham International for collaboration and Squibb & Sons, Ltd. for a gift of hydroxyurea.

**References**

ACKERBLOM, L., EHRENBERG, A., GRASLUND, A., LANKINEN, H., REICHARD, P. & THELANDER, L. (1981). Overproduction of the free radical of ribonucleotide reductase in hydroxyurea-resistant mouse fibroblast. *Proc. Natl Acad. Sci. USA*, **78**, 2159.

ARIEL, I.M. (1970). Therapeutic effects of hydroxyurea: experience with 118 patients with inoperable tumours. *Cancer*, **25**, 705

BAGSHAWE, K.D. (1985). Cancer drug targeting, *Clin. Radiol.*, **36**, 545.

BAGSHAWE, K.D. (1986). Reverse role chemotherapy for resistant cancer, *Lancet* **ii**, 778.

BENSON, A.B., TRUMP, D.L., CUMMINGS, K.B. & FISCHER, P.H. (1985). Modulation of 5-iodo-2′-deoxyuridine metabolism and cytotoxicity in human bladder cancer cells by fluoropyrin. *Biochem. Pharmacol.*, **34**, 3925.

CURT, G.A., CLENDENNIN, N.J. & CHABNER, B.A. (1984). Drug resistance in cancer. *Cancer Chemotherapy Rep.*, **68**, 87.

DJORDJEVIC, B. & SZYBALSKI, W. (1960). Genetics of human cell lines: III Incorporation of 5-bromo and 5-iodo deoxyuridine into the deoxyribonucleic acid of human cells and its effect on radiation sensitivity. *J. Exp. Med.*, **112**, 509.

FISCHER, P.H., VASQUEZ-PADUA, M.A., REZNIKOFF, C.A. & RATSCHAM, W.J. (1986). Preferential stimulation of iododeoxyuridine phosphosylation by 5′-aminothymidine in human bladder cancer cells *in vivo*. *Cancer Res.*, **46**, 4222.

GOLDIE, J.H. & COLDMAN, A.J. (1984). Genetic instability in the development of drug resistance. *Sem. Oncol.*, **12**, 222.

HAMPTON, E.G. & EIDINOFF, M.L. (1961). Administration of 5-iododeoxyuridine-$I^{131}$ in the mouse and rats. *Cancer Res.*, **21**, 345.

HOFER, K.G. (1980). *Toxicity of radionuclides as a function of subcellular dose distribution*, p. 371. Third International Radiodosimetry Symposium, US Dept. of Health and Human Services.

LEMOTTE, P.K. & LITTLE, J.B. (1984). DNA damage induced in human diploid cells by decay of incorporated radionuclides. *Cancer Res.*, **44**, 1307.

LEWENSOHN, R., RINGBORG, U. & HANSSON, J. (1982). Different activities of unscheduled DNA synthesis in human melanoma and bone marrow cells. *Cancer Res.*, **42**, 84.

PRUSOFF, W.H. (1959). Synthesis and biological activities of iododeoxyuridine, an analog of thymidine. *Biochem. Biophys. Acta*, **32**, 295.

PRUSOFF, W.H. (1963). A review of some aspects of 5-iododeoxyuridine and azauridine. *Cancer Research*, **23**, 1246.

SCHUHMACHER, J., KAMPMANN, H., MATTERN, J., VOLM, M., WAYSS, K. & ZIMMERER, J. (1974). Incorporation of $^{131}$iododeoxyuridine into the DNA of tumour-bearing rats after partial synchronisation as a tool in scintigraphic tumour localisation. *J. Nucl. Med.*, **12**, 309.

SNEIDER, T.W. & POTTER, V.R. (1969). Alternative *de novo* and 'salvage' pathways to thymidine triphosphate synthesis: possible implications for cancer chemotherapy. *Cancer Res.*, **29**, 2398.

TATTERSALL, M.H.N. & HARRAP, K.R. (1973). Changes in the deoxyribonucleoside triphosphate pools of mouse 5178Y lymphoma cells following exposure to methotrexate or 5-fluorouracil. *Cancer Res.*, **33**, 3086.

TAYLOR, G.A., JACKMAN, A., CALVERT, A.H. & HARRAP, K.R. (1983). Plasma nucleoside and base levels following treatment with the new thymidilate synthetase inhibitor (B3717). In *Purine Metabolism in Man*, **IV**, Part B, Biochemical, Immunological & Cancer Res., p. 379. Plenum Press: New York.